# Ureteric Trauma following Stent Removal in Kidney Transplant Recipient: A Unique Case of Prolonged Morbidity

**DOI:** 10.1155/2021/9959074

**Published:** 2021-04-30

**Authors:** Ahmad Mirza, Imran Gani, Andy Shi Huang, Ravi Mallavarapu, Laura Mulloy, Muhammad Saeed, Rajan Kapoor

**Affiliations:** Department of Transplant, Augusta University Medical Center, Medical College of Georgia, 1120 15th Street, AD, 3401 Augusta, Georgia

## Abstract

A 52-year-old African-American male patient with end-stage renal disease due to hypertension underwent deceased donor kidney transplant procedure with no immediate complications. The postprocedure complications, interventions, and course were abstracted by chart review. The ureteric stent was removed with flexible cystoscopy on postoperative day (POD) 24. 24 hours later, the patient presented with abdominal pain and inability to urinate. An urgent ultrasound and noncontrast CT scan showed grade 4 hydronephrosis of the transplanted kidney. A percutaneous nephrostomy stent was placed for urinary diversion. A large ureteric hematoma filling the lumen of the mid to distal ureter was identified on the nephrostogram and was evacuated. A follow-up nephrostogram on POD 44 revealed a distal ureter stricture and persistent well-formed midureter filling defect. A repeat nephrostogram performed at POD 72 was done with stricture dilatation, internalization of stents, and removal of a percutaneous nephrostomy tube. The patient was maintained on antibiotics for UTI prophylaxis throughout the course.

## 1. Introduction

Kidney transplantation is the only intervention that improves overall morbidity and mortality in patients with end-stage renal disease (ESRD) [1]. More than 19,000 kidney transplants were performed in United States in 2017 [2]. There has been a significant advancement in immunosuppression medication, which has contributed to the overall improvement in renal transplant graft survival [3]. The surgical technique for vascular anastomosis has remained largely unchanged, and for index renal transplant procedures, the vascular anastomosis is performed with the external iliac artery and vein [4]. The early posttransplant surgical complications have a significant impact on graft survival, leading to increased long-term morbidity and mortality. The incidence of ureteric complications after transplant ranges from 1 to 15% [5, 6]. Several studies have addressed the issue of urological complications and have identified stent deployment as a possible explanation of decreased incidence.

In most transplant centres, it is a routine clinical practice to use ureteral stents for ureter to bladder anastomosis during kidney transplant with removal after 3 to 4 weeks with flexible cystoscopy [5, 7]. The stent removal procedure is nontraumatic, and complications during removal are rare but can happen. We report and discuss an unusual complication of ureteral stent removal in a renal transplant recipient, which resulted in prolonged morbidity and repeated interventions.

## 2. Case Report

**Figure 1:**
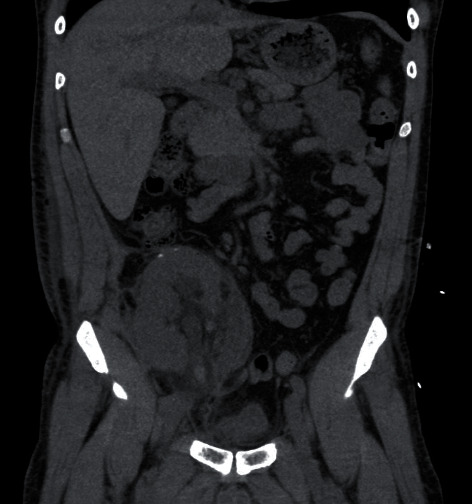
Coronal view of the CT scan showing hydronephrosis and clot in the transplanted ureter.

**Figure 2:**
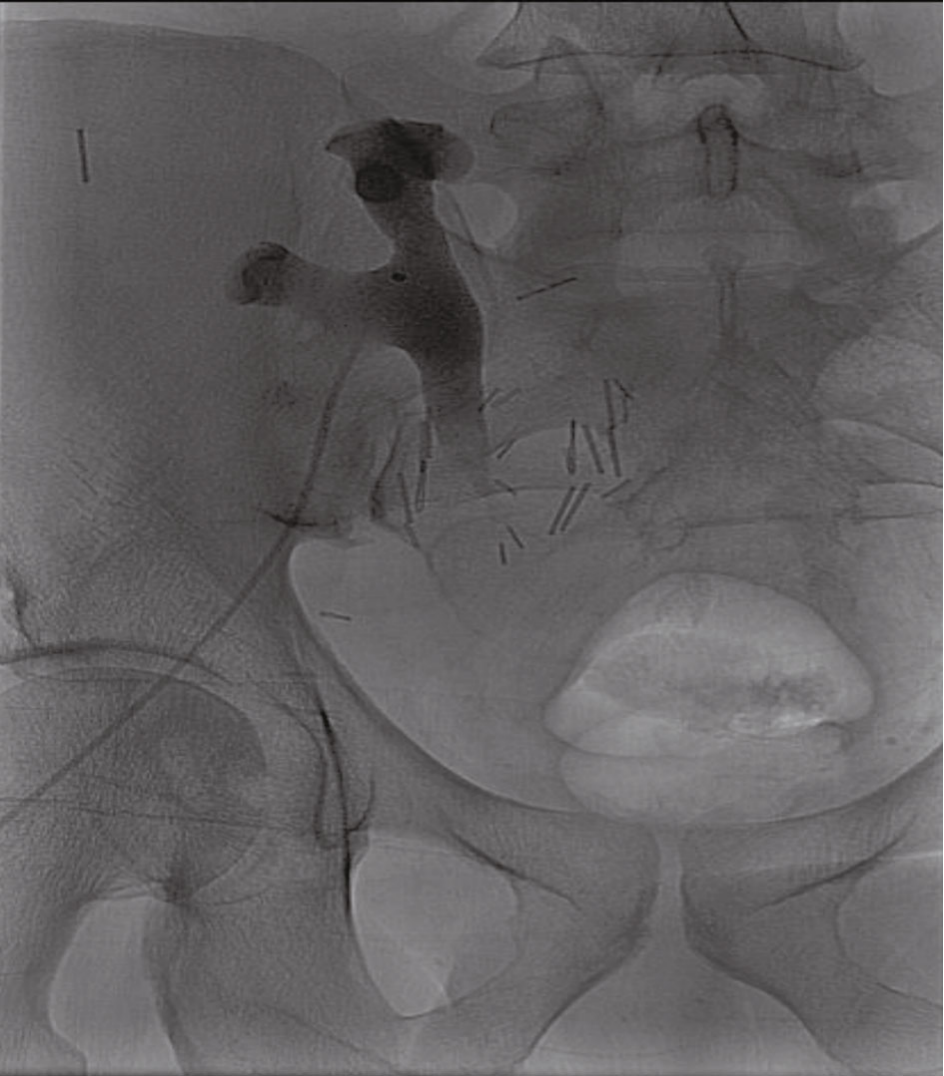
Postnephrostogram on readmission with no distal flow of contrast in the ureter.

**Figure 3:**
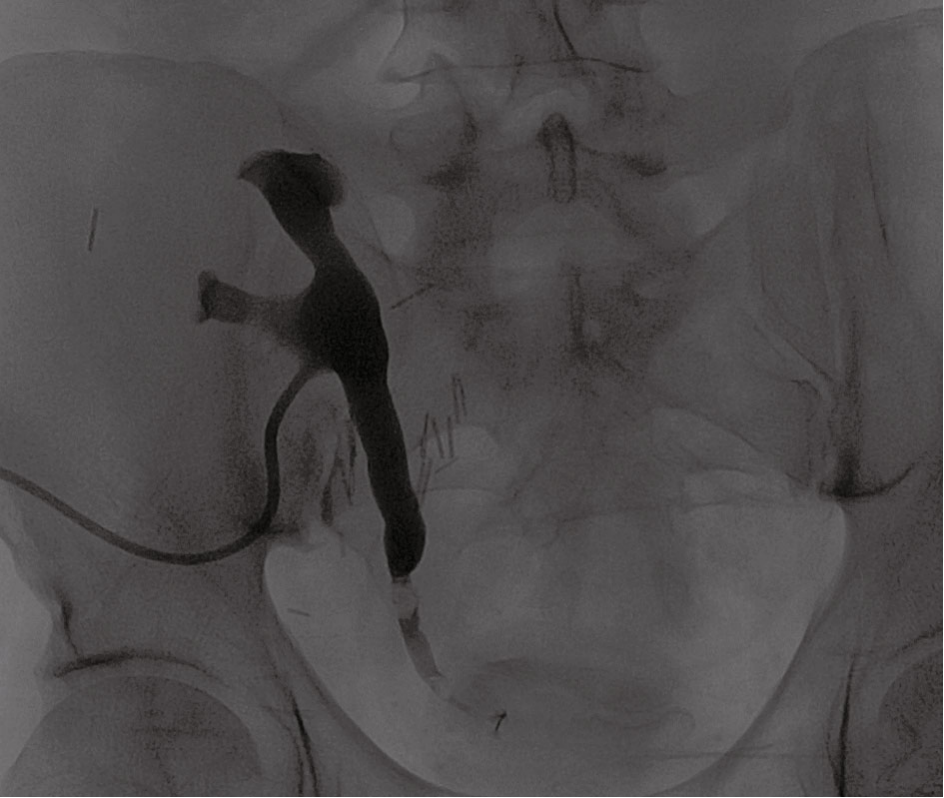
Posttransplant day 44 nephrostogram with mid to distal ureter stricture and clot presence.

**Figure 4:**
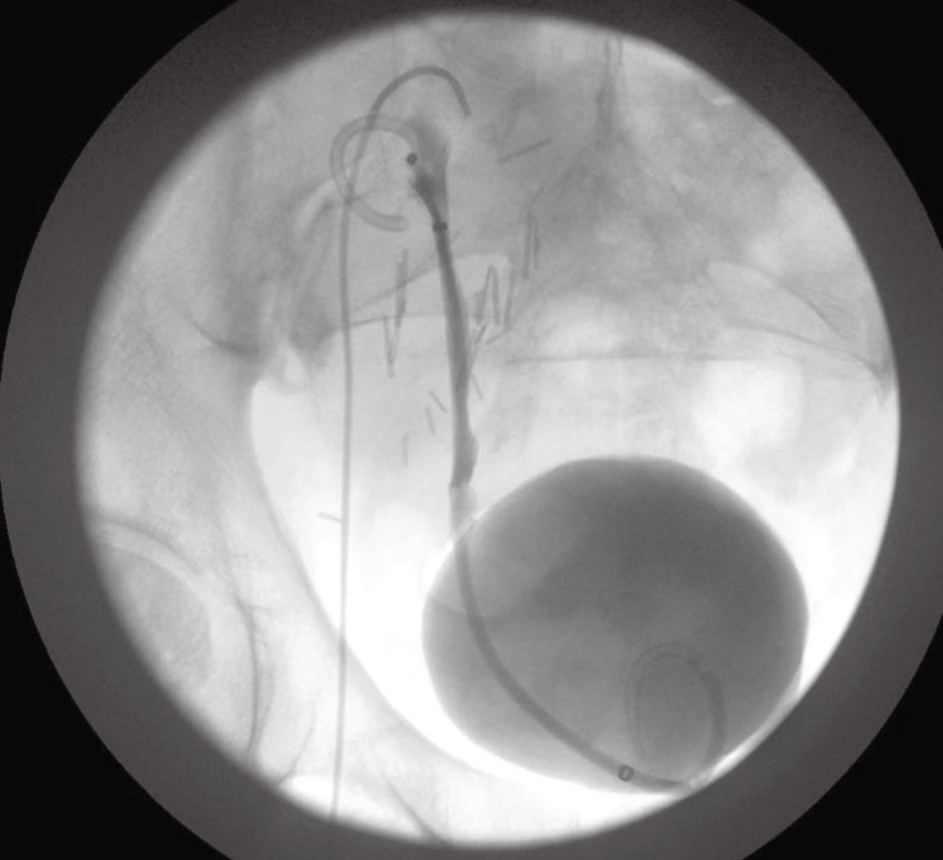
Posttransplant day 72 nephrostogram with placement of double J stents and removal of percutaneous nephrostomy tube with free flow of contrast in the bladder lumen.

A 53-year-old African-American male patient with end-stage renal disease due to hypertension underwent deceased donor kidney transplant from a 49-year-old donor. Both virtual and physical cross-match were performed before the transplant. The Lich-Gregoir antireflux method for ureteroneocystostomy was performed. The procedure was uncomplicated, and the patient was discharged on postopt day 5. As induction, immunosuppressive therapy standard 3 doses of thymoglobulin (1.5 mg/kg) were administered. He was started on tacrolimus, mycophenolate mofetil, and steroids as maintenance immunosuppression. On postopt day (POD) 24, he underwent scheduled removal of the ureteric stent with flexible cystoscopy. The patient was discharged soon after the procedure. On POD 26, two days after the removal of the ureteric stent, he presented with abdominal pain, anuria, and acute kidney injury. His creatinine was 4.20 mg/dl from 2.94 mg/dl a day earlier. An immediate ultrasound showed significant hydronephrosis of the transplanted kidney with an increase in resistive indices. A noncontrast CT scan of the abdomen and pelvis was suggestive of a blood clot mid to distal ureter and a large collection around the transplanted kidney (Figure 1). Imaging results were consistent with obstructive uropathy. The patient immediately underwent percutaneous drainage of seroma (triglyceride and creatinine levels did not reflect lymphocele or urinoma) and an urgent nephrostogram along with percutaneous nephroureteral stent placement (PCNU) (Figure 2). On postprocedure day 1, PCNU was capped and patient voided. The patient initially complained of hematuria which gradually improved during the course of four-day inpatient stay. Serum creatinine decreased appropriately. On the day of discharge, patient denied pain and was ambulating and voiding. On POD 37, the patient underwent a repeat nephrostogram, and PCNU was converted to a percutaneous nephrostomy tube. On POD 44, the patient underwent a repeat nephrostogram for tube exchange and aspiration of ureteral filling defects. Well-formed blood clots were removed by employing suction and basket extraction technique, and also a distal ureter stricture was identified (Figure 3). On POD 72, the patient underwent a repeat IR nephrostogram with balloon dilatation of mid to distal ureteral stricture and placement of a double-J ureteral stent (Figure 4). Again, a large blood clot was retrieved from the transplanted ureter. Nephrostomy tube was removed with the internalization of stent. The patient has since recovered with the above complication and procedures contributing to prolonged morbidity. The allograft is functional with baseline creatinine and urine output. The patient continues on oral antibiotics for the prevention of infection. We reviewed the cause for initial ureteric injury and most likely it resulted from the kinking of the stent inside the ureter, and during removal with cystoscopy, it resulted in laceration of the ureteric epithelium causing hematoma formation and blocking the urine outflow.

## 3. Discussion

During the earlier experience with kidney transplantation, the ureterovesical anastomosis was performed by employing the Leadbetter-Politano method. However, this has mostly been replaced by the Lich-Gregoir antireflux method for ureteroneocystostomy [8–10]. Stenting of the ureter anastomosis with the bladder has been practiced with the rational of decreasing the incidence of urine leak, stenosis, and promoting urine drainage [7, 11]. Despite the reported benefits, several studies have associated significant morbidity with the routine use of stents [12–14]. Earlier literature identified little benefit in the routine placement of ureteric stents [15, 16]. However, subsequent studies reported significant clinical benefit from routine use of stents [17, 18]. These findings were also related to advancement in the material of stent used with least adhesive nature and use of peri-procedural antibiotics leading to a decrease in the incidence of urinary tract infections [16].

Sansolone et al. reported the largest series of review of practice for routine placement of ureteric stents during kidney transplant [19]. The study reported an increased incidence of urine leak and stenosis when stents were not used. Also, the use of stents was not associated with increased hematuria and urinary tract infections. In the majority of transplanted patients, the stents are asymptomatic. The routine insertion of ureteric stents is supported by a large meta-analysis, which also identified their association with decreased incidence of ureter infections [18]. A randomized control trail of stent versus no-stent reported an overall decrease in complications (15%), urine leak (8%), and obstruction (0%) if stents were used [11]. The trial also recommended the removal of stents within 4 weeks of transplant.

The insertion of a stent is linked with few minor complications of dysuria, increased frequency, hematuria, suprapubic, and loin discomfort. The major complications described include stent migration and increased vesicoureteral reflux causing hydronephrosis, fracturing of stent, and ureteral necrosis resulting in partial or complete disruption of anastomosis [20]. Ureteral stent removal is generally a safe procedure. However, it is important to be aware of stent migration and kinking which can predispose to complications at removal. Ureteric stents are removed by flexible cystoscopy in a retrograde fashion. Retrograde retrieval can be challenging in case of stent encrustation, previous surgery on the bladder, proximal migration of the stent, inability to maintain lithotomy position, or enlargement of the prostate. A history of surgery resulting in an inaccessible retrograde route, urethral stricture, fragmentation of the proximal stent, and inability to find the ureteral orifice with a cystoscope are other challenging scenarios. An antegrade approach through a percutaneous nephrostomy may be an alternative for retrieval of a ureteral stent in case the retrograde approach fails. However, it is challenging or is not routinely feasible.

A Cochrane review of five studies concluded ureteric stenting decreased rate of urinary complications by 6% (OR 0.24, *p* = 0.02) [21]. Another meta-analysis reported decrease of 7.5% in urinary complications in patients undergoing routine ureteric stenting [18]. Significant cost saving with routine stenting from prevention of urological complications has been identified in transplant recipients [22].

The stents in the transplanted ureter are asymptomatic. Patients are not aware of the stents and do not routinely complain of dysuria or hematuria unless there is a super-added infection. Benoit et al. randomized control trial between stented and nonstented transplant patients showed the same frequency of urinary tract infections between the two groups [23]. There is a reported advantage of stents in promoting healing of anastomosis site, facilitating urine drainage, and preventing kinking of ureter from peri-transplant fluid collections [20].

The double J stents which are deployed during ureter and bladder anastomosis during kidney transplant are generally safe and effective. In the literature, their use has effectively decreased the urological complications of urine leak, stenosis, and fistula formation [19]. Their early removal within four weeks is advocated to reduce the incidence of stent migration and urinary tract infections. They are associated with minor and few rare major complications. Generally, their use is safe and improves the graft outcome.

## Data Availability

No data were used to support this study. The figures used to support the findings of this study are included with the article.
